# Location-Allocation and Accessibility Models for Improving the Spatial Planning of Public Health Services

**DOI:** 10.1371/journal.pone.0119190

**Published:** 2015-03-16

**Authors:** Gina Polo, C. Mera Acosta, Fernando Ferreira, Ricardo Augusto Dias

**Affiliations:** 1 Laboratory of Epidemiology and Biostatistics. Department of Preventive Veterinary Medicine and Animal Health. University of São Paulo, São Paulo, Brasil; 2 Institute of Physics. University of São Paulo, São Paulo, Brasil; University of Florida, UNITED STATES

## Abstract

This study integrated accessibility and location-allocation models in geographic information systems as a proposed strategy to improve the spatial planning of public health services. To estimate the spatial accessibility, we modified the two-step floating catchment area (2SFCA) model with a different impedance function, a Gaussian weight for competition among service sites, a friction coefficient, distances along a street network based on the Dijkstra’s algorithm and by performing a vectorial analysis. To check the accuracy of the strategy, we used the data from the public sterilization program for the dogs and cats of Bogot´a, Colombia. Since the proposed strategy is independent of the service, it could also be applied to any other public intervention when the capacity of the service is known. The results of the accessibility model were consistent with the sterilization program data, revealing that the western, central and northern zones are the most isolated areas under the sterilization program. Spatial accessibility improvement was sought by relocating the sterilization sites using the maximum coverage with finite demand and the p-median models. The relocation proposed by the maximum coverage model more effectively maximized the spatial accessibility to the sterilization service given the non-uniform distribution of the populations of dogs and cats throughout the city. The implementation of the proposed strategy would provide direct benefits by improving the effectiveness of different public health interventions and the use of financial and human resources.

## Introduction

The integration of location-allocation and accessibility models provide a framework for investigating the use of the health care services and for generating alternatives either to suggest an efficient service or to improve an existing one [1]. Accessibility to health care services refers to the relative ease with which health care can be reached from a given location [2–4]. Many factors may influence access to health care services, such as the availability of health sites in the area (supply), the population size in the area (demand) and the geographic barriers between supply and demand, among others [5].

Several methods have been proposed to estimate spatial accessibility: the regional availability model [6], kernel density models [2], the gravity model [7] and floating catchment area models [8]. A number of studies have employed the two-step floating catchment area (2SFCA) model first proposed by Radke and Mu [9] to estimate spatial access to human health care services [2, 3, 10, 11]. This method works in two steps: 1) Estimate the demand for each service site within a threshold distance from the catchment area and calculate the site-to-population ratio according to the site’s capacity and the local demand. 2) Sum the ratios of the services sites within a threshold distance of the catchment of each population centroid. This method assumes that all the population locations within a catchment area have equal impedance while disregarding differences in accessibility within the catchment [8]. Luo and Qi [12] considered for each point a travel time catchment area divided into three sub-zones, assuming equal access for all the population locations within a sub-zone. Polo, Mera and Dias [13] integrated a different Gaussian function into the 2SFCA model to progressively discount the access within a catchment area. Thus, two relatively neighboring points could have different accessibility values, which could not be observed when working with sub-zones. Albeit this modification does not present the difficulties of the previous models, it has limitations because it does not consider changes in the population’s demand when an individual or population centroid has access to more than one service site. In this regard, Wan, Zou and Sternberg [14] integrated a competition weight based on the travel time to each service site that assumes that the population demand for a particular service is influenced by the availability of other neighboring services.

Although spatial accessibility models allow the evaluation of public health care services, they are unable to suggest the allocation or reallocation of these services. The integration of location-allocation models with accessibility models in geographic information systems address this limitation and allow the generation of different location-relocation alternatives. Spatial location-allocation models have been used to assist the planning of public and private services [1]. The minimum impedance and maximum limited coverage models are the most efficient models for planning public services [15]. Some examples include Eaton [16] who used the maximum coverage problem to allocate clinics in a rural area of Colombia, Rahman and Smith [1] who allocated health services in Bangladesh and Eaton and colleagues [17] who studied the problem of allocating ambulances in the Dominican Republic.

In the present study we integrated location-allocation and spatial accessibility models in geographic information systems as a comprehensive strategy proposal for assisting in the spatial planning of public health care services and for facilitating the population’s accessibility to different public health interventions. The proposed methodology was evaluated using the data from the public sterilization program for the dogs and cats of Bogotá, Colombia but it is expected that it could be applied to other public health interventions.

## Materials and Methods

### Spatial accessibility model

To estimate spatial accessibility we developed a model based on the two-step floating catchment area (2SFCA) model of Radke and Mu [9]. The first step of the model is to calculate a competition weight *W*_*ij*_ between the population *i* and the health care sites *j* [14]. A matrix is created with the distances from the population *i* to any service site *k* within the catchment delimited by *d*_0_. Each component of the matrix situated within the area *d*_0_ is assigned a competition Gaussian weight *W*:

$$W_{ij}=\frac{T_{ij}}{\;\sum_{k\in \{d_{ik}<d_{0}\}}T_{ik}}$$ (1)

where *d*_*ik*_ is the travel distance from the population *i* to any service site *k* within the catchment delimited by *d*_0_ and *T*_*ij*_ and *T*_*ik*_ are the assigned Gaussian weights for *j* and *k*, respectively in relation to the population *i*. This competition weight assumes that the demand for a service site is affected by the proximity of other health care sites. Thus, for a population *i*, there will be greater accessibility among more service sites in this coverage area due to decreased demand at each service site. The *W*_*ij*_ will be equal to 1 if only one service site is in the catchment area, but decreases as the number of available alternatives increase.

The second step, is to integrated a Gaussian impedance function *G*_(*d*_*ij*_, *d*_0_)_, to each component of the matrix. The impedance function used was the previously proposed by Dai [5] because it has a gradual decay over long distances than the other functions [5].

The Dai Gaussian weight is expressed as:

$$\begin{array}{c} G\left(d_{ij},d_{0}\right)=\left\{\begin{array}{cc} \frac{e^{-\beta {\left(d_{ij}/d_{0}\right)}^{2}}-e^{-\beta }}{1-e^{-\beta }}, & \text{if}\;d_{ij}\leq 0\\ 0, & \text{if}\;d_{ij}>0 \end{array}\right. \end{array}$$ (2)

where *β* in this paper is the coefficient of friction representing the difficulty of pedestrian displacement for the road conditions [18]. We set *β* = 0.5, which is a conservative value for an ordinary pedestrian walking under many foreseeable conditions [19].

The third step is to calculate the health care-to-population ratios *R*_*j*_. The population at *i* weighted with a Gaussian function *G*_(*d*_*ij*_, *d*_0_)_ within the catchment *d*_0_ of *j* represents the potential users of the health care sites. The ratio *R*_*j*_ is given by:

$$R_{j}=\frac{S_{j}}{\sum_{i\in (d_{ij}\leq d_{0})}P_{i}G(d_{ij},d_{0})W_{ij}}$$ (3)

where *P*_*i*_ is the population at location *i* within the catchment *d*_0_, *S*_*j*_ is the capacity (i.e., number of surgeries, vaccine doses, physicians, hospital beds) of the health care site *j*, *d*_*ij*_ is the travel distance between a population location *i* and a health care site *j* and *G*(*d*_*ij*_, *d*_0_) is the Gaussian impedance function.

The fourth step is to search all sterilization sites *j* within the threshold distance *d*_0_ for each population location *i*. After subtracting each *R*_*j*_ using the Gaussian function *G*(*d*_*ij*_, *d*_0_), the health care-to-population ratios *R*_*j*_ are summed to obtain the spatial accessibility at population location *i*:

$$\begin{array}{c} A_{i}=\sum_{i\in (d_{ij}\leq d_{0})}W_{ij}R_{j}G\left(d_{ij},d_{0}\right) \end{array}$$ (4)

where the annotations are the same as in Eq.(3).

The major advantage of our accessibility model over that proposed by Radke and Mu [9] lies in the application of a vectorial analysis, a Gaussian weight for competition among service sites, network distances based on the Dijkstra’s algorithm and a more accurate impedance function with a friction coefficient, which allows different spatial accessibility for each population site depending on the surrounding facilities and the travel pattern in accessing the service sites. The maps were made using the Network Analyst Tool of ArcGis 10.1 (Environmental System Research Institute, Inc., Redlands, CA).

### Location-allocation models

Although this study did not create or modify a location-allocation model, it demonstrated the possibility of integrating these models in geographic information systems with other interaction models. In this paper we applied the minimum impedance or *p*-median and the maximum coverage with finite demand models [15].

#### Minimum impedance or *p*-median model

In this model, for a given demand, the number *p* of facilities is calculated to minimize the total of the weights of the transportation distances between facilities and demand [15]. This assumes that the service users use the nearest facility. The *p*-median model can be expressed as: minimize

$$\begin{array}{c} Z=\sum_{i}\sum_{j}\alpha_{i}d_{ij}x_{ij} \end{array}$$ (5)

subject to:

$$\begin{array}{c} \sum_{j}x_{ij}=1\;\forall i, \end{array}$$

$$\begin{array}{c} x_{ij}\leq y_{j},\;\forall i,j, \end{array}$$

$$\begin{array}{c} \sum_{j}y_{j}=p, \end{array}$$

$$\begin{array}{c} x_{ij},y_{i}\in \left\{0,1\right\}. \end{array}$$

where *x*_*ij*_ = 1 if demand *i* is assigned to a facility *j* and *x*_*ij*_ = 0 otherwise, *n* is the number of demand sites, *α*_*i*_ is the population of demand *i*, *d*_*ij*_ is the shortest distance between *i* and *j* and *p* is the number of facilities to be located.

#### Maximum coverage with finite demand model

This model seeks to maximize the population that can be serviced within a threshold distance for a number of service locations [15]. The facilities are chosen so that all or most of the demand is within the distance limit *S*. This model is: maximize

$$\begin{array}{c} z=\sum_{i\in I}\alpha_{i}y_{i} \end{array}$$ (6)

subject to:

$$\begin{array}{c} \sum_{j\in N_{i}}x_{j}\geq y_{i},\;\forall i, \end{array}$$

$$\begin{array}{c} \sum_{j\in J}^{n}x_{j}=P, \end{array}$$

$$\begin{array}{c} x_{j}=\left(0,1\right)\;\forall j, \end{array}$$

$$\begin{array}{c} y_{j}=\left(0,1\right)\;\forall i, \end{array}$$

where *I* denotes the set of demand nodes, *J* denote the set of facility sites, *x*_*j*_ = 1 if a facility is located at *j* and *x*_*j*_ = 0 otherwise, *y*_*i*_ = 1 if demand from *i* is covered by a facility and *y*_*i*_ = 0 otherwise, *N*_*i*_ = {*j*∣*d*_*ij*_ ≤ *S*} is the set of facilities which are eligible to provide cover for demand *i*, *S* is the distance beyond which a demand point is considered uncovered, *d*_*ij*_ is the shortest distance between *i* and *j*, *p* is the number of facilities to be located and *α*_*i*_ is the population to be served at demand *i*.

### Strategy evaluation

**Fig 1:**
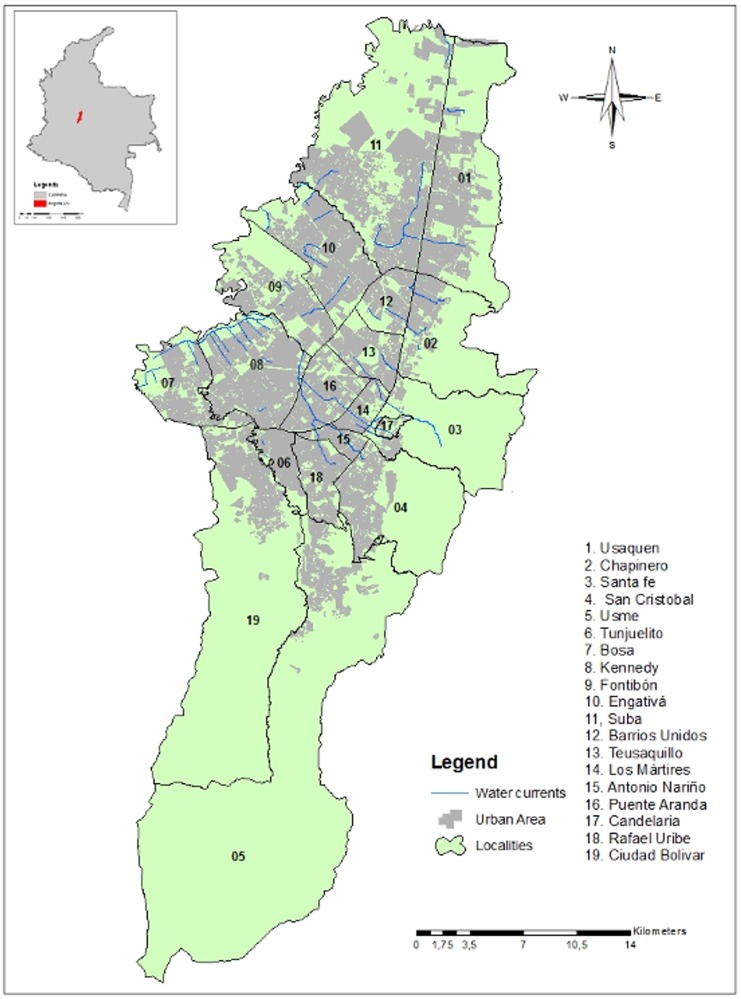
Urban area and water barriers of Bogotá, Colombia. The 19 localities of the urban area and all the water barriers were considered in the accessibility and location-allocation analysis.

The integration of the location-allocation and the accessibility models was evaluated using the data of the public sterilization program for the dogs and cats of the city of Bogotá from December 2010 to November 2011. Although the accuracy of the strategy was checked using this data, it could be applied to any public health intervention if the required parameters are known. This study area is located in central Colombia and has 7,363,782 inhabitants [20], 631,276 dogs and 131,344 cats in the urban area [21]. The city is divided into 20 localities, 19 of which are in the urban area (Fig. 1). The sterilization program for dogs and cats in the city is a public health intervention critical for the population management of dogs and cats and for the control of urban rabies. The District Department of Health through the Center for Zoonosis Control and the public hospitals of primary level conduct the program using mobile units offering free surgical sterilizations throughout the city. In 2011, 22,981 surgeries were performed on 13,275 dogs and 9,706 cats by 257 mobile units [22]. The capacity of the mobile units ranged from 14 to 1,275 sterilizations, with an average of 310 sterilizations per site per year [22]. For the accessibility model the populations of dogs and cats were geographically located using the georeferenced layer of the households of the city obtained from the Spatial Data Infrastructure for the Capital (IDECA) [23]. We calculated the number of households with dogs and cats using the estimation of the dog and cat populations conducted by Vega, Espinosa and Castillo [21].

## Results and Discussion

In order to show that the integration of the location-allocation and the accessibility model is suitable to improve the spatial planning of public health services, we used the data from the public sterilization program for the dogs and cats of Bogotá, Colombia. However, in the proposed strategy, the only variable associated with the health service was the capacity of each site. Since we used the data of a sterilization program, the capacity of the service was the number of surgeries per site, but it could be any other, such as vaccine doses, hospital beds, number of physicians, etc., depending on the type of health service to be assessed. As such, the suggested strategy is independent of the service and can be applied if the service capacity is known.

**Fig 2:**
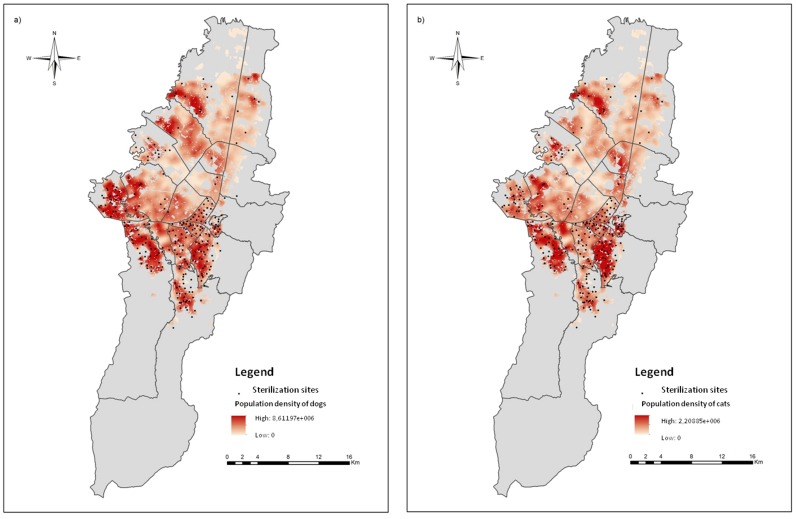
Spatial distribution of the sterilization sites. a. Sterilization sites in relation to the population density of dogs. b. Sterilization sites in relation to the population density of cats.

The 257 addresses of the dog and cat sterilization sites from December 2010 to November 2011, distributed among the 19 localities that compose the urban area of Bogotá, were manually georeferenced to ensure the accuracy of the procedure. Fig. 2 shows the spatial distribution of the sterilization sites in relation to the dog and cat population density. There is a low concentration of sterilization sites in the north of the city where there are areas of low population densities of dogs and cats. The central and western zones have few sterilization sites and low animal density. The highest number of sterilization sites is found in the southern and southeastern zones, coinciding with zones of high densities of dogs and cats.

**Fig 3:**
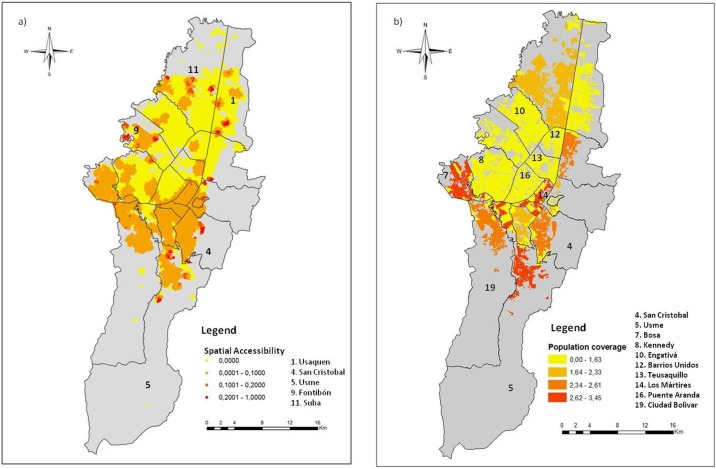
Comparison between the spatial accessibility using the model developed in this paper and the percentage of the population covered in each locality, obtained from the data of the public sterilization program for dogs and cats. a. Spatial accessibility of the population to the sterilization program of dogs and cats. b. Population coverage of the sterilization program for dogs and cats calculated using the data of the public service from December 2010 to November 2011.

Fig. 3A shows the spatial accessibility of the dog and cat population to the sterilization sites using the model developed in this paper. Lower accessibility was observed in the central and northern zones. The southern part of the city, corresponding to the area with the highest number of sterilization sites, showed better accessibility to the program. Small areas with high spatial accessibility were observed in the localities of Usaquen, Suba, Fontibón, Usme and San Cristobal, as indicated in Fig. 3A. These areas of high spatial accessibility in the localities of Usme, Fontibón and San Cristobal coincide with a high density of sterilization sites and in the localities of Usaquen and Suba correspond to low animal density zones.

Using the estimations of the dog and cat populations of Vega, Espinosa and Castillo [21] and the data of the mobile sterilization units, it was estimated that from December 2010 to November 2011 the public sterilization program covered 2.10% of the dog and 7.38% of the cat populations [22]. The representation of the percentage of the population covered in each locality, obtained from the data of the public sterilization program for dogs and cats from December 2010 to November 2011, is found in Fig. 3B. The spatial pattern of the results of the accessibility model (Fig. 3A) matches with the spatial pattern of the population coverage obtained from the real data of the public service (Fig. 3B). As an example, Kennedy, Engativá, Barrios Unidos, Teusaquillo and Puente Aranda in the western and the central part of the city, had the lowest sterilization proportion and also very low accessibility, while San Cristobal, Usme, Bosa, Los Mártires and Ciudad Bolivar in the south and south-central, had the highest proportion of sterilized dogs and cats and showed higher spatial accessibility. Thus, the results obtained from the analysis of the local data agree with those obtained from the accessibility model. Moreover, red areas in Fig. 3B correspond to a large number of service sites as shown in Fig. 2, however, in Fig. 3A these areas do not exhibit high spatial accessibility because the service capacity is exceed by the high animal population density (Fig. 2). These results suggest that the accessibility model provides an accurate approximation to the real use of the services and can serve as a basis for the evaluation, planning and reformulation of public health programs.

**Fig 4:**
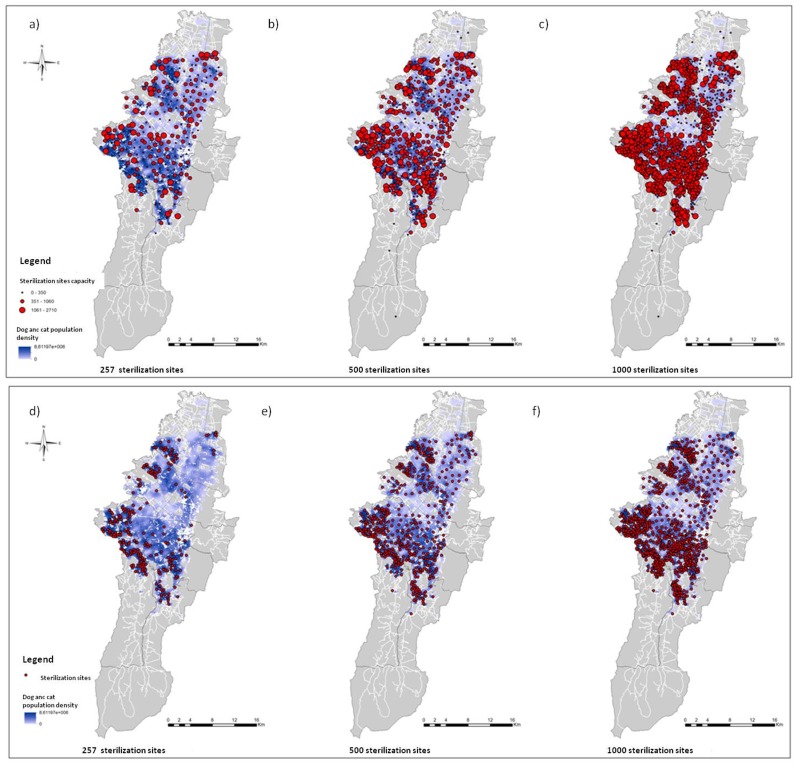
Result of the location-allocation model. a, b, c. Using the p-median model with different amounts of sterilization sites: 257 (current number), 500 and 1000, respectively. d, e, f. Using the maximum coverage model with a capacity of 310 surgeries per sterilization site.

Because the current spatial location of the sterilization service provides inadequate spatial accessibility and an inadequate distribution of resources (Fig. 3A), we created six different scenarios by varying the spatial distribution, the capacity of the services and the number of sterilization sites by using the maximum coverage with finite demand and the *p* median location-allocation models (Fig. 4). The scenarios were based on different service capacities and three different quantities of sterilization sites: 257 (current number), 500 (twice the current number) and 1,000 (fourfold the current number). In the maximum coverage model, the capacity was fixed at 310 sterilizations per site, the actual average capacity [22]. The locations of the sterilization sites were selected from a number of places of interest where the sterilization program and other public health services are provided. These places included local municipalities, district centers of attention (CADES), public medical care centers (CAMI), public schools, community centers, community hospitals, churches and parks.

**Fig 5:**
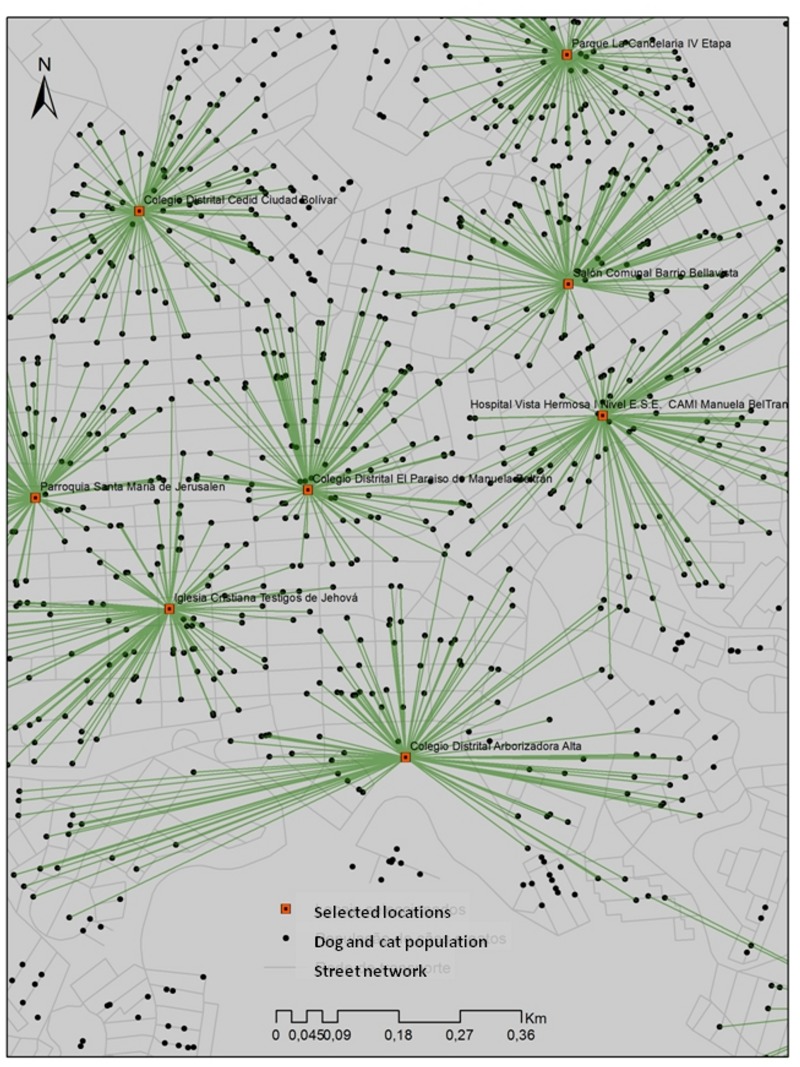
Enlargement of some selected locations in the location-allocation models.

Comparing the results of the location-allocation models, the distributions of the sterilization sites are different with regard to both the solution used and the number of sterilization sites considered in each scenario (Fig. 4). Using the maximum impedance model (Figs. 4A, 4B and 4C), the sterilization sites are dispersed uniformly in the area and each site has a different capacity. Fig. 4A shows the scenario with the existing number of sterilization sites, Fig. 4B with twice the number of existing sterilization sites and Fig. 4C with fourfold the actual number of sterilization sites. Figs. 4D, 4E and 4F show the results of the maximum coverage model, with the sterilization sites concentrated in the areas with greater animal population density (demand). Fig. 4D illustrates the maximum coverage model using the actual number of the sterilization sites, Fig. 4E shows twice the number of sites and Fig. 4F shows fourfold the actual number of sites. Fig. 5 enlarges a portion of a selected location in the locality of Ciudad Bolivar, displaying some of Ciudad Bolivar’s public schools, a public hospital of Vista Hermosa and Catholic churches.

**Fig 6:**
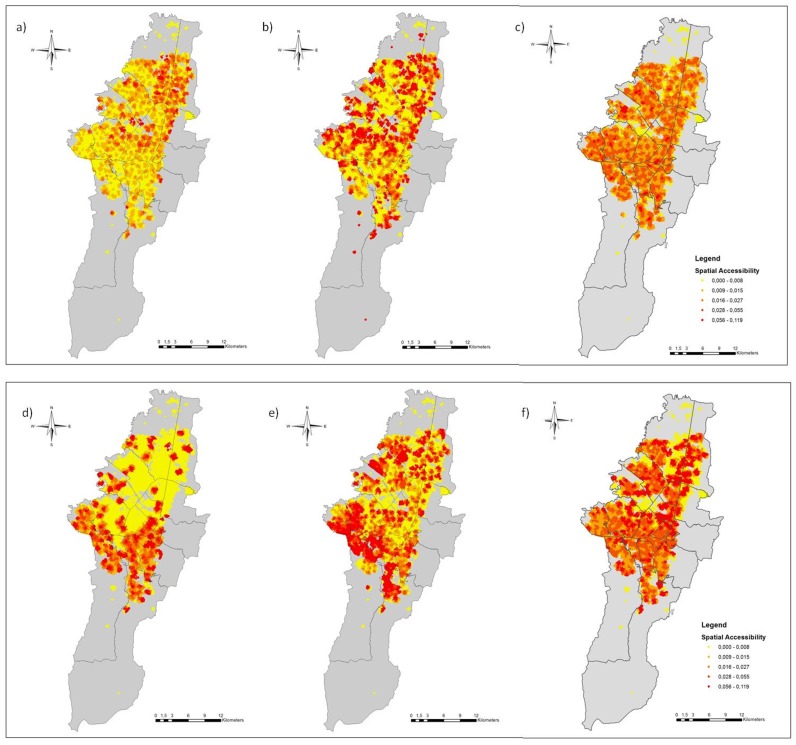
Spatial accessibility to the sterilization sites arising from the location allocation models. a, b, c. Using the p-median model with different amounts of sterilization sites: 257 (current number), 500 and 1000, respectively. d, e, f. Using the maximum coverage model with a capacity of 310 surgeries per sterilization site.

A spatial accessibility analysis was performed to evaluate the effectiveness of the allocation strategy. Fig. 6 shows the spatial accessibility to the sterilization sites from the location-allocation solutions. In the two models, the spatial accessibility to service increase as the number of points increases. Figs. 6A and 6D exhibit the spatial accessibility of the *p*-median model and the maximum coverage model, respectively, using the current number of sterilization sites. The spatial accessibility derived from the maximum coverage model of Fig. 6D is greater than the spatial accessibility obtained from the current location of the sterilization program of Fig. 3A, optimizing the spatial access in areas of high population demand with the same number of sterilization sites. In contrast, in Fig. 6A because of the uniform distribution of the points, the more favored zones are those with the lowest population densities of dogs and cats located mainly in the north and central areas of the city. Hence, the *p*-median model is ineffective given that it overlooks the distribution of the dog and cat populations in the city (Figs. 4A, 4B and 4C) and provides a lower spatial accessibility (Figs. 6A, 6B and 6C). In contrast, the maximum coverage model was the best adapted to the non-uniform distribution of the dog and cat population because it maximized the health service accessibility in areas of high population density (Figs. 6D, 6E and 6F).

According to the Bogotá mobility census, people walk on average 15 minutes per day [24]. Moreover, a standard human reach a walking speed of 4 kilometer per hour on level ground [25]. For that reason, the impedance threshold distance *d*_0_ used in the present work was 1,000 meters. Nonetheless, a travel distance *d*_0_ of 800 meters has also been used in previous health research and is considered as a reasonable walking distance based on empirical studies [13, 26]. This threshold distances may present a limitation in rural areas, given that the distance threshold for these areas is greater [27]. Additionally, this study contemplates a strategy for pedestrians because, according to the secretary of mobility of Bogotá, 46% of the trips that are made in the city are by walking [24]. This value is greater than that of other types of transport, including public collective transport and private car, the latter comprising only 10% of the total trips in the city [24]. Another advantage of developing a pedestrian model is the production of a more precise analysis because it is independent of the traffic and the direction of the roads. Consideration of other means of transport, such as private cars and public transport, could give a more complete understanding of spatial accessibility to different services.

In some cities, the sterilization of dogs and cats has not had the desired results because of low sterilization rates, but when rates of 70% were achieved, the population size was reduced [28]. Ferreira [29] concluded that permanent sterilization programs have decreasing costs and that significant reductions in the population density may be achieved in four to five years, depending on the sterilization rates applied (i.e., 0.80 years^−1^). We found that it is possible to increase the population coverage of public health services by strategically allocating the sites in the areas of high population density, as is shown in Figs. 6D, 6E and 6F for the sterilization program for dogs and cats. In our particular case, the public service of Bogotá must consider the cost-benefit of either increasing the number of sites or increasing the capacity of the points. Additionally, public service of Bogotá should obtain analogous results for other public health intervention offered to the community.

## Conclusion

The results demonstrate that the integration of spatial accessibility and location-allocation models represent an alternative solution for problems associated with the spatial planning and the unequal distribution of public health services that could not be addressed with models of spatial accessibility only. The principal advantage of the suggested strategy is the consideration of different spatial accessibility for each population site depending on the surrounding facilities and the travel pattern in accessing the service sites. Since the proposed strategy is independent of the service, it has an important policy implication for the planning of different health programs in the city of Bogotá, Colombia, and potentially other services in other countries worldwide.

## Supporting Information

S1 Spatial Datasets All spatial datasets of the dog and cat sterilization program of Bogotá, Colombia, necessary for the performance of the methodology presented in the paper. The datasets are in shapefile format and storing the geometric location and the attribute information of the geographic features used: sterilization sites, dog and cat population, localities, places of interest, streets and barriers.
(ZIP)
